# Supplementary material for: Are carbon emissions trading and green financial instruments synergistic? -Comprehensive quantitative research based on content analysis
Source: PLoS One. 2024 Mar 7;19(3):e0298601. doi: 10.1371/journal.pone.0298601 (PMC10919601; doi:10.1371/journal.pone.0298601)
Supplement: S1 Data — (ZIP) [file pone.0298601.s001.zip › Data/地级市Z_F工作报告.zip!/┴╔─■/╜⌡╓▌╩╨/└┤╫╘╣1⁄2╓┌║┼í╛┬φ┐╦╩2╛▌═°í┐_2012.docx]

各位代表：

　　现在，我代表市政府向大会报告工作，请予审议，并请市政协各位委员提出意见和建议。

一、2011年工作回顾

　　2011年，在省委、省政府和市委的正确领导下，我们坚持以科学发展为主题，以转变经济发展方式为主线，锐意进取，真抓实干，全面完成了市十四届人大四次会议确定的各项任务，实现了“十二五”的良好开局。

　　按可比口径初步预计，全市实现地区生产总值1100亿元，增长13.5%；固定资产投资660亿元，增长32%；地方财政一般预算收入103.6亿元，增长27.8%；规模以上工业增加值530亿元，增长15.8%；社会消费品零售总额374亿元，增长17.5%；实际利用外资6亿美元，增长20%；引进域外资金700亿元，增长75%；出口总额14.5亿美元，增长22.9%；城市居民人均可支配收入20155元，增长16%；农民人均纯收入9307元，增长20%。经济结构进一步优化，三次产业比重调整为16∶49∶35。实现了全年“两个不低于”的奋斗目标，辽宁沿海第二大城市建设迈出坚实步伐。

　　(一)沿海经济带建设实现新突破

　　抢抓实施辽宁沿海经济带开发开放国家战略和举办2013中国·锦州世界园林博览会等历史机遇，强力推进基础设施建设和产业发展，沿海大开发大开放格局全面拉开。

　　世园会筹建取得阶段性成果。总体规划、修建性详细规划获得审批；土石方回填、微地形、园区基础设施建设和大树栽植等园区生态环境建设基本完工；展园及标志性建筑、近背景区改造等工程建设全面展开；招商招展、宣传策划工作全面启动。广大建设者在攻坚克难中凝聚成的“锦州世园精神”，正激励着全市上下戮力同心，确保举办一届成功、精彩、难忘的“海上世园会”。

　　“双亿吨”大港建设取得重大进展。锦州港实现货物吞吐量7200万吨，增长19.9%；集装箱运输80万标箱，增长14.2%。中电投煤炭码头一期工程进展顺利。龙栖湾港区规划调整全部完成，防波堤、围堰、2个5万吨级泊位及航道工程建设快速推进。口岸大厦建成并投入使用。

　　开发区西海工业园区基础设施建设不断完善，石油储备基地、中信金属15万吨高碳铬铁等一批项目开工建设，汽车及零部件、农产品加工等产业集群进一步壮大，临港产业初具规模。滨海体育中心、职教园区等项目加快推进。白沙湾生活区公共服务能力日益增强。

　　龙栖湾新区总体规划、控制性详细规划及专项规划基本完成。道路管网、景观绿化等基础设施建设步伐加快。“澳海澜庭”等地产项目开工建设，“中国海洋城”建设开始起步。奥克阳光、阳光能源等15个项目开工建设；中国光伏材料产品质量检测检验中心正式挂牌，锦州被命名为国家硅材料高新技术产业化基地；差别化纤维一期20万吨项目顺利推进，光伏、化纤纺织“双千亿”产业基地建设取得明显成效。

　　建业经济区管理机构正式成立，总体规划通过专家评审。

　　大有经济区起步区基本实现“七通一平”，科诚电阻器、光华航模发动机等重大项目开工建设。

　　(二)工业五项工程建设迈出新步伐

　　预计全年完成工业固定资产投资325亿元，增长67.5%；共实施亿元以上重点工业项目73个，规模以上工业企业达到727户。

　　项目年活动收效明显。辽宁抚挖460台大吨位起重机、石化公司天然气制氢装置等24个项目竣工投产；石化公司25万吨烷基化装置、惠发天合50万吨船用油等32个项目开工建设；天桥钼业1500吨钼酸铵、3万吨氯化法钛白粉等技改扩能项目顺利实施。光伏、钛及特种金属、汽车及零部件、精细化工等8大产业集群不断壮大，产值均突破100亿元，其中光伏产值突破150亿元。新增主营业务收入10亿元以上企业4户。完成并购项目6项。规模以上工业增加值能耗下降6.1%。

　　科技创新体系进一步完善，企业自主创新能力快速提高。完成专利申请557件，新认定国家高新技术企业3家，27项科技项目列入国家和省科技计划，组建省级工程技术研究中心2家。引进海外研发团队7项。规模以上高新技术产品增加值增长35%。

　　(三)县域经济发展实现新跨越

　　县域新一轮“双增升位”计划开局良好，四县(市)完成地区生产总值560亿元，增长13.8%；地方财政一般预算收入43.5亿元，增长32.2%；固定资产投资320亿元，增长32.5%。

　　工业园区基础设施建设和产业集群发展同步推进。四县(市)新增园区基础设施建设投资12亿元、标准厂房85.6万平方米，建成区面积达到22.9平方公里。重点园区主导产业基本确立，累计新引进项目389个，总投资359亿元。

　　农业结构调整步伐加快。粮食总产量突破50亿斤，肉蛋奶总产量突破140万吨。新增设施农业面积8万亩、产业化龙头企业50家，引进和推广农业新品种、新技术91项，订单农业达到240万亩，“三品”认证面积达到270万亩。义县辉山国大乳业、北镇五峰谷物、凌海宏基园菊苣等8个投资10亿元以上的农产品加工项目开工建设。农业“一县一业”基本确立。

　　农村生产生活条件不断改善。节水滴灌增收工程全面启动。土地开发利用和耕地保护卓有成效。气象预报预警、防灾减灾能力进一步提高。耕种收综合机械化水平达到67%。连续6年无重大动物疫情和畜产品安全事件发生。完成小流域治理27.3万亩。解决了10.35万人饮水安全问题。8.74万农村贫困人口人均增收600元。新建农村客运站5座，新增农村公路里程330公里。建成新农村现代流通网络工程网点120个、户用沼气池1万个。县域房地产施工面积达到240万平方米。

　　(四)城区经济和服务业发展实现新提升

　　推动了城区下放管理权限和区划调整两项改革，城区发展活力日益显现，第一个城区经济“三年倍增”计划全面完成。古塔、凌河、太和三城区完成地区生产总值370亿元，增长13%；地方财政一般预算收入26.2亿元，增长33%；固定资产投资140亿元，增长33%。

　　服务业聚集区建设势头良好，中央大街十里商街服务业聚集区被评为省级示范区，古塔区商贸流通聚集区、辽西小商品·古玩商贸流通聚集区被省政府认定为首批省级现代服务业聚集区。亿隆国际、温州名购、茂业百货、地一大道等重大服务业项目建设快速推进，太阳广场、站东、安乐里等城市综合体项目前期工作全面展开。喜来登酒店、国际会展中心建成投入运营。

　　中国(锦州)北方农业新品种新技术展销会、中国(锦州)古玩文化节等展会品牌日臻成熟。锦州机场年旅客吞吐量突破10万人次。凌河紫荆圣泉度假山庄等3个温泉旅游项目取得实质性进展。旅游业总收入185.5亿元，增长53.8%。全市实现房地产投资110亿元、商品房销售额143亿元。金融机构新增贷款120亿元。新华龙钼业、锦州银行、五峰米业、光和密封等企业上市工作进展顺利。

　　(五)改革开放取得新进展

　　国企改革实现新突破，86户企业改制任务基本完成，安置职工3.32万人。文化院团改革全面启动。集体林权配套改革稳步实施。医药卫生体制改革整体推进，基层医疗卫生机构综合改革取得阶段性成果并通过省验收。

　　积极开展招商引资活动，中国光伏之都、中央大街十里商街、宁波温泉旅游、香港新城新市镇建设等主题概念招商和主导产业招商活动收效明显。成功举办了第一届中国(锦州)国际光伏产业博览会，签约协议金额232亿元。第四届中蒙八地区经济合作交流会在锦召开，与蒙古国签署了铁路领域战略合作框架协议。国家加工贸易梯度转移重点承接地建设全面推进。加大对重点出口企业扶持力度，确保了外贸出口的平稳较快增长。

　　(六)生态环境和基础设施建设再上新台阶

　　“迎世博绿家园”活动和青山保护工程全面启动。实施城市街路、绿地加密覆盖工程，新增城市绿地15.6万平方米。渤海大道、龙栖湾大道、南站地区绿化亮化改造工程效果明显。凌河流域生态化治理取得阶段性成果，完成封育面积17.5万亩。全市完成造林绿化53.8万亩。北镇市获得全省首个省级生态市殊荣。并网改造小锅炉22台。新增集中供热面积220万平方米，城市集中供热率达到85%。8座污水处理厂稳定运行。4条出市境河流断面监测达标率100%。城区空气质量优良率达到96%。

　　锦凌水库截流工程成功合龙，枢纽工程初具规模；建成移民安置新村3个。锦州湾机场项目进入国家审批阶段，飞行区地基处理与填筑试验工程通过验收。城市轨道交通项目筹建工作扎实推进。凌海大道、生活垃圾及污泥焚烧发电厂等项目开工建设。广州街公铁立交桥实现简易通行。中央南街等16条街路新建大修工程如期完工。

　　(七)重点民生工程取得新成效

　　创业型城市创建工作稳步推进，普惠制就业培训4.8万人，实名制安置就业5.6万人，零就业家庭始终保持动态为零，登记失业困难家庭高校毕业生就业率达到100%，城镇登记失业率控制在3.6%以内。社会保险扩面工作任务超额完成。城镇居民养老保险和新型农村养老保险全面实施。连续6年保持农民工工资无年度内新生拖欠，清理拖欠农民工工资偿还率达到100%。

　　保障性安居工程完成1810套公租房、1800套廉租房、520套经济适用住房、9926套棚改房及1060套农垦危房改造任务，全面完成省政府下达的建设指标。城乡低保和各类优抚对象抚恤补助提标工作顺利完成。新建、扩建民办养老机构8所，新增养老床位612张。新增城市社区办公用房1万平方米，建成农村社区服务站40个。

　　全面实施教育发展规划，大力推进校安工程和平安校园建设，新建改造校舍3万平方米，全面消除D级危房。新改扩建农村幼儿园21所，全市学前3年入园率达到89%。成功举办了“中国锦州笔架山海洋音乐节”、“红诗红歌、唱响锦州”等大型群众文化活动。76个乡镇文化站建成投入使用。道光廿五、辽西木偶等4个非遗项目赴台湾展示展演。新建农民健身示范广场30个。网羽中心竣工投入运营。

　　卫生信息化建设全面推进，5家医疗机构建立电子病历。康宁医院完成整体搬迁，4所县(市)医院建成投入使用。新农合筹资标准提升到年人均230元。人口计生综合改革取得实质性进展，计划生育家庭保障水平明显提高。

　　扎实推进社会管理创新，公安信息化建设、社会治安防控体系建设取得重大突破，城区警务改革经验全省推广，群众安全感指数名列全省前茅。“五五”普法成果显著，“六五”普法全面启动，乡镇法律援助站(点)覆盖率达到100%。平抑市场物价，生活消费品价格水平总体保持稳定。深入开展矛盾纠纷排查，狠抓源头预防化解，群众合理诉求得到有效解决。社会稳定、安全生产、消防、食品药品安全等工作进一步加强，和谐锦州建设迈出新的步伐。

　　(八)政府机关作风建设实现新转变

　　自觉接受人大法律监督、政协民主监督和群众的社会监督，坚持定期向人大和政协报告、通报工作，积极采纳各民主党派、工商联、无党派人士意见。全年共办理市人大代表建议和政协提案517件，办复率、满意率达100%。

　　行政执法监督和政务公开力度加大，行政权力运行进一步规范。全面推行全程办事代理制，行政审批制度改革不断深化。坚持重大项目投资跟踪审计，资金使用效益明显提高。“五大系统”建设全面展开，政府机关作风建设进一步加强。

　　拥军优属、军民共建活动深入开展，实现省级双拥模范城“七连冠”。国防动员、民兵预备役工作成绩显著。民族团结事业不断向前推进。对口援建工作扎实开展。国家安全、妇女儿童、应急管理、外事侨务、防震减灾工作不断加强。统计、质监、档案、残联、慈善等工作取得新的成绩。

　　各位代表，回顾过去一年的工作，我们深切感到，这些成绩的取得，是省委、省政府和市委正确领导的结果，是全市广大干部群众共同努力奋斗的结果。在此，我代表市政府，向在各个领域和岗位上辛勤劳动、做出贡献的全市人民表示崇高的敬意！向给予政府工作大力支持的人大代表、政协委员、各民主党派、工商联和各人民团体，向关心和支持政府工作的离退休老同志，向中省直各部门、各单位，向驻锦人民解放军和武警部队官兵，向所有关心支持锦州发展的海内外朋友，表示衷心的感谢！

　　在总结成绩的同时，我们也清醒地看到，当前锦州经济社会发展还面临一些困难和问题。一是经济实力不够强，县域和城区经济总量还低于全省平均水平，与建设辽宁沿海第二大城市的要求还不相适应；二是个别县(市)区主导产业定位还不够明晰，产业集群规模不大，优势不明显；三是经济结构不尽合理，服务业在三次产业中的占比偏低，经济外向度不高，具有牵动作用的龙头企业、重大项目不多；四是软环境建设还存在一些不容忽视的问题，政府自身建设还有待进一步加强。这些困难和问题，都需要在今后的工作中切实解决。

二、2012年工作安排

　　今年是实施“十二五”规划的关键之年，是世园会建设的攻坚之年，也是实现“三年倍增”的决战之年。扎实做好今年工作，对于加速推进辽宁沿海第二大城市建设具有重要意义。

　　今年政府工作的总体要求是：全面贯彻落实党的十七届六中全会、中央和省经济工作会议、市委十一届二次全会精神，准确把握“稳中求进、稳中求快”的总基调，以科学发展为主题，以转变经济发展方式为主线，全面实施沿海突破战略，加快推进沿海、县域、城区三大经济区域协调发展，不断开创科学发展、创新发展、和谐发展的新局面，为建设辽宁沿海第二大城市奠定更加坚实的基础。

　　全市经济社会发展的主要预期目标是：地区生产总值增长13.5%；固定资产投资增长30%；地方财政一般预算收入增长20%；规模以上工业增加值增长15%；社会消费品零售总额增长17%；实际利用外资增长20%；引进域外资金增长10%；出口总额不低于全省平均增幅；城市居民人均可支配收入增长16%；农民人均纯收入增长17%。

　　今年，国际经济会持续低迷，国内经济增速也将趋缓，我们面临的国内外经济形势将更加严峻复杂。但这种严峻复杂的经济形势，也恰恰是我们加快发展的重大机遇、调整结构的重大机遇、扩大对外开放的重大机遇。只要我们紧紧抓住机遇、积极应对挑战，就完全可以战胜困难、逆势上扬，全面实现发展目标。在实际工作中，我们要咬定目标不放松，确保实现一季度“开门红”、上半年“双过半”和全年“两个不低于”，继续保持经济平稳较快发展的良好态势。

　　按照以上要求和目标，我们要重点抓好以下八个方面的工作：

　　(一)以世园会建设为重点，深入实施沿海突破战略

　　充分发挥实施沿海开发开放国家战略、国家和省级开发区等政策优势，以世园会建设为牵动，强化规划引领，推进体制机制创新，全面优化沿海经济区发展空间和产业布局，努力实现产业集群和城市功能协调互动发展，加快形成沿海经济隆起带和沿海城市带。

　　举全市之力加快世园会建设。继续发扬攻坚克难、敢打硬仗的“锦州世园精神”，全面完成基础设施、标志性建筑、80个展园以及园区生态环境建设工程，统筹推进园区各类商业资源开发、大型文化演艺活动策划、市场宣传推介等工作。龙栖湾大道、渤海大道背景区景观带要基本建成，污水处理厂、安保中心、公交中心要投入使用，确保全面具备开园基础条件。深入开展“我为世园做贡献”全民行动，全面提高市民文明素质，加快形成人人关心世园会、人人参与世园会、人人奉献世园会的浓厚氛围。

　　加大锦州港基础设施建设力度，开工建设25万吨级航道、华润电力煤码头和油品罐区，确保三港池3个通用泊位投入试运行，实现港口吞吐量8000万吨、集装箱运输90万标箱。积极推进龙栖湾港区3万吨级航道、防波堤、一港池通用泊位等工程建设，确保2个泊位具备简易通航条件。

　　强力推进开发区汽车及零部件、农产品加工等产业和中北石化润滑油等一批重点项目建设，促进产业集群发展，做大临港工业。开工建设365互联网客服基地、白沙湾国际温泉休闲度假区、滨海新城医院等项目，加快滨海体育中心、职教园区、商贸中心、五星级酒店等项目建设进程，全面提升城市服务功能。实施白台子村、崔庄子村、孙家湾村整体搬迁，切实抓好锦港大街等8条街路绿化改造及滨海路景观带建设，树立滨海新区崭新形象。

　　加速推进“中国海洋城”和“双千亿”产业基地建设，全面提升龙栖湾新区形象。深化同西部控股等基金公司的合作，推进土石方回填、道路管网、绿化亮化及世园会背景区基础设施建设，开工建设总部基地、渤海湾1号、海洋巨蛋、五星级酒店等10个项目和客运枢纽、邰滨线一级公路工程，确保旭龙太阳能30兆瓦太阳能电池、差别化纤维一期20万吨等17个项目竣工投产。

　　建立健全建业经济区管理体制和运行机制，探索建立公司制、市场化运作模式。进一步完善航空物流城空间和产业规划，加快推进起步区基础设施建设，开工建设锦州湾机场、航空物流城主干路和机场防护堤工程。

　　全面推进大有经济区基础设施和配套设施建设，突出抓好产业转移承接地示范园区建设，引领和带动其他工业园区在承接产业转移上有更大作为。

　　(二)以产业集群建设为重点，深入实施工业五项工程

　　全年计划安排5亿元以上工业项目32个，总投资575亿元。其中新开工10个，总投资100亿元。

　　重点推进光伏和化纤纺织“双千亿”产业基地、钛及特种金属、石油精细化工四大产业集群建设，积极培育汽车及零部件、新型冶金材料、大型工业炉、农副产品深加工等产业集群。力促奥克阳光500兆瓦多晶硅片、北镇辽宁华美达铜业10万吨无氧铜杆等续建项目竣工投产，确保锦州钛业6万吨氯化法钛白粉、义县众德广润300万吨短流程连铸等项目开工建设。新增主营业务收入10亿元以上企业7-8户。完成并购项目6项。规模以上工业增加值能耗下降5%以上。积极推进公用事业企业和厂办大集体企业改革，做好86户企业资产处置及遗留问题处理工作。

　　充分发挥我市科研院所、高等院校聚集的优势，实施关键技术攻关与高新技术产业化工程，加快将松山高新技术产业园区打造成为国家级高新技术产业园区和国家科技成果转化服务示范基地，促进科技成果向新兴产业转化。完成专利申请565项。开发新产品580种。争取4-5户企业被认定为省级技术中心、30个项目列入省技术创新重点项目。引进海外研发团队6项。规模以上高新技术产品增加值增长35%。

　　(三)以“三化”建设为重点，推动县域经济跨越发展

　　咬定新一轮县域经济“双增升位”计划不动摇，坚持工业化、城镇化、农业产业化同步推进，落实扶持政策，下放管理权限，狠抓要素保障，加快壮大县域经济整体实力。

　　加快县域工业化进程。全面提升县域工业园区基础设施建设水平，建立完善公共服务平台，提高项目承载能力。加大招商引资力度，推进产业集群发展，确保四县(市)新上规模以上项目100个，每个县(市)至少打造1个产值超百亿元的工业产业集群。

　　加快县域城镇化进程。统筹规划县城和中心镇建设，加快实施黑山“两城一区”、北镇广宁新区和沟帮子河西新区、凌海凌河新城、义县宜州新城等新城新市镇建设，加大城中村、城边村改造力度，确保四县(市)城镇化率提高2个百分点。

　　加快农业产业化进程。重点抓好义县辉山国大乳业、北镇五峰谷物等8个投资10亿元以上重大农产品加工项目建设，尽快投产达效。开工建设黑山励家杂粮加工物流园、北镇广宁台湾绿色农产品深加工产业园、义县万华秸秆板材和水产养殖加工等4个10亿元以上农产品加工项目。新增规模以上农业产业化龙头企业50户。进一步做强做大优势特色产业，力争有1-2个县(市)成为省“一县一业”示范县。

　　继续实施凌河保护、辽河治理和青山保护工程，全面完成省年度任务。加强现代畜牧业示范区建设，切实做好重大动物疫情防控和畜产品安全监管工作。进一步提高气象预报预警、防灾减灾能力。新增节水滴灌面积13万亩、水浇地面积15万亩、设施农业面积8万亩、造林绿化面积56.1万亩。耕种收综合机械化水平达到69%。

　　全力做好土地供应工作，推进增减挂钩项目区拆旧进程，扩大周转用地指标，加大对批而未用、低效使用和废弃地的处置力度，最大限度盘活存量土地。

　　(四)以服务业聚集区建设为重点，加快城区经济和服务业发展

　　大力实施新一轮城区经济“三年倍增”计划，进一步向城区下放经济管理权限，激发城区经济发展活力，形成支撑和带动服务业发展的新增长点，提高服务业在三次产业中的占比。

　　全力打造中央大街十里商街等现代服务业聚集区，加快茂业百货、亿隆国际、温州名购、国际五金机电城等在建项目建设进程，开工建设太阳广场、安乐里、站东、红星美凯龙等大型城市综合体项目。加快推进国际烧烤美食总汇等重点项目开工建设，力促台湾百脑汇IT总部大厦、SK信源农产品物流等一批重大项目早日开工建设。

　　全面繁荣城乡消费市场，建设商品配送中心2-3个、新农村现代流通网络工程网点150个，改造信息化连锁农家店500家。大力发展会展经济，精心办好光伏产业博览会、农展会、古玩文化节等各类展会。确保房地产开发投资和商品房销售额分别增长15%以上。

　　整合旅游资源，强化宣传促销，加快医巫闾山、北普陀山文化旅游项目和紫荆圣泉度假山庄等温泉旅游项目建设，推进一批宾馆酒店和景区提档升级，努力实现旅游业大发展和城市形象大提升。

　　拓宽融资渠道，搭建合作平台，确保筹措发展建设资金300亿元。积极推进光大银行等域外银行在锦设立分支机构。大力支持农村信用社、村镇银行、小额贷款公司和融资担保机构发展，组建小额贷款公司10家、融资担保公司5家。加快企业上市步伐，确保新增上市企业2户。

　　(五)以体制机制创新为重点，全面扩大对内对外开放

　　适应国际国内经济发展形势的新变化，全面加大招商引资工作力度，加快构建全方位、多层次、宽领域的对内对外开放新格局。

　　创新招商引资体制机制，设立市级招商直属分局和部门分局，实施重大项目领导包扶、亿元以上项目备案制度，制定落实新的优惠政策和奖励办法，层层放大招商工作平台，尽快形成全民参与招商引资的浓厚氛围。

　　有效整合配置现有项目资源，大力推进以商招商，切实抓好面向长三角、珠三角的主题概念招商和产业集群招商。全面做好欧美、日韩、香港、台湾等国家和地区的专题招商，确保在实际利用外资方面取得更大的突破。

　　充分利用国家加工贸易梯度转移重点承接地建设这一历史机遇，进一步明确各园区产业定位，加快基础设施和配套设施建设，全面提高项目承接能力，吸引更多的东南沿海企业聚焦锦州、投资锦州。

　　加强石化、铁合金等6大出口基地建设，大力扶持机电、高科技、装备制造业产品和农产品出口，积极培育新的出口增长点，不断扩大出口规模，努力实现出口创汇的新突破。

　　(六)以城市规划为重点，加速提升锦州城市建设管理水平

　　牢固树立规划是重要战略资源的理念，坚持规划先行。抓紧整合提升城市总体规划、沿海经济带总体规划及重要功能节点的规划设计水平。统筹老城区和沿海经济带的公共服务设施和基础设施建设规划，全面提升重要发展空间的公共服务水平和项目承接能力。进一步完善城市地空规划和人防工程建设规划。围绕小凌河、女儿河、北普陀山、南山等得天独厚的自然生态资源，重点做好小凌河、女儿河城区段的生态环境治理规划，做好锦凌水库、中央大街十里商街、松山新区等重要空间节点和功能区域的规划，加快城市“南扩”、“东联”、“西进”、“北展”进程，进一步提高城市品位，提升空间价值。

　　全力抓好重大基础设施和重点城建项目建设。城市轨道交通项目要全面开工建设。确保锦凌水库主体工程如期竣工，全面完成混凝土坝、桥梁、闸门安装等建设任务和库区移民安置年度工作任务。加快推进锦赤铁路建设，开工建设中央南街光彩立交桥、广州街小凌河大桥、单洞扩孔和解放路东扩工程。确保凌海大道、阜盘高速公路锦州段工程竣工通车。新建女儿河“南湖公园”，全面打造“两河”滨河景观带。完成16条城区主干道和宝锦线、大锦线等公路大修改造工程。全面推进供热计量改革。并网改造小锅炉11台。全年96%以上天数空气质量达到国家二级标准。

　　围绕世园会建设，加快推进国家园林生态城市、国家卫生城市和全国环保模范城市创建工作，全面加强城市综合整治。深入实施城市绿化、亮化、美化、净化和道路畅通工程，全面提高住宿、餐饮、商业、娱乐等设施的服务保障水平，努力实现城市面貌的大改观和接待能力的大提升。

　　(七)以改善保障民生为重点，推进社会事业全面发展

　　时刻关注人民群众安危冷暖，确保改革成果更多惠及民生，更好地提升人民群众的幸福感。

　　大力实施民生工程。深入开展创业型城市创建工作，推进创业培训、创业服务，实施普惠制培训3万人以上，实名制安置就业4万人，确保城镇登记失业率控制在3.6%以内。强力推进“五大社会保险”扩面征缴工作，平均净增参保人数1.2万人以上，新型农村养老保险和城镇居民养老保险参保覆盖率达到85%以上。全面实行居民医保门诊统筹，启动实施事业单位工伤、生育保险工作。加快推进养老服务社会化进程，新增养老床位500张。实施新一轮扶贫攻坚计划，确保10.8万农村低收入群体年人均收入超过2200元。解决农村7万人饮水安全问题。开工建设城市新水源地。继续采取各种有效措施稳定物价。加快推进残疾人社会保障体系和服务体系建设。全面完成保障性住房建设任务，努力使中低收入家庭住有所居。

　　全面发展社会事业。切实加强基础教育，各县(市)区全部通过省“双高普九”验收，力争有1-2个县(市)区进入全省首批基础教育强县(市)区行列。加大职业教育专业布局调整，组建5个职业教育集团。强力推进新建小区配套建设幼儿园工作。加大医疗资源整合力度，建设公共卫生中心和五官医院。全面推进卫生信息化建设，实现就医“一卡通”。深化人口和计划生育综合改革，建立面向家庭、全社会、全人口的服务新模式。加强“一乡一站”建设，推进乡镇文化站提档升级。实现市、县“三馆”免费开放。以辽西古玩城改扩建为重点，新建5个文化产业园区，促进文化产业聚集发展，做大做强文化产业。高标准做好第十二届全运会羽毛球、拳击比赛项目筹备工作，举办锦州市第一届全民健身运动大会。积极稳妥推进事业单位分类改革。切实抓好县级公立医院综合改革试点工作。加快实施新华书店转企改制、国有文艺院团改革、文化广电管理体制改革和文化市场综合执法改革，着手筹建锦州演艺集团。切实做好统计“一套表”改革工作。

　　推动社会管理创新。加强社会治安防控体系建设，完善城区警务改革，深化农村警务改革，全面提升公安信息化管理效能。继续保持严厉打击各类刑事犯罪的高压态势，不断增强人民群众的安全感和满意度。加大公安基础设施建设，推进公安工作科学发展。促进军政军民团结，开展第八轮双拥模范城争创工作。加强民族宗教工作，巩固民族团结和谐局面。全面推进“六五”普法，深化普法依法治理。认真落实领导包案和信访接待日制度，有效化解各类社会矛盾。建立监管长效机制，继续抓好生产安全、消防安全、食品药品安全等工作，全力维护锦州社会稳定和政治安定。

　　(八)以提升行政效能为重点，全面加强政府自身建设

　　坚持依法行政、科学民主决策，自觉接受人大及其常委会的法律监督、工作监督，积极支持政协履行政治协商、民主监督和参政议政职能，坚持重大事项向人大报告和向政协通报制度，坚决执行人大及其常委会的各项决议、决定，认真办理人大议案、代表建议和政协提案。

　　全面推进“五大系统”建设，积极推进行政权力公开透明运行，不断扩大人民群众的知情权和参与权。进一步加大审计监督工作力度。大力开展“软环境建设服务年”活动，启动实施第五次行政审批制度改革，建设公共行政服务中心，不断优化经济发展环境，全面提升行政服务水平。

　　建立坚强有力的行政指挥系统。大力倡导事必躬亲、事无巨细、事不过夜的工作作风，始终满怀发展激情，勇于担当，敢于决策，全面提高行政理解力和执行力，善于破解发展难题，确保各项工作有着落、有结果。切实加强公务员队伍素质和能力建设，建立健全鼓励先进、鞭策后进、奖勤罚懒的责任目标体系，努力塑造务实高效、风清气正的政府形象。

　　各位代表，实施沿海突破战略，建设辽宁沿海第二大城市的进军号角已经吹响，任重道远，催人奋进；全面完成今年各项工作，任务艰巨，使命光荣。让我们在省委、省政府和市委的坚强领导下，在市人大、市政协的监督支持下，紧紧团结和依靠全市人民，让一切劳动、知识、技术、管理、资本的活力竞相迸发，让一切创造社会财富的源泉充分涌流，为建设富庶文明幸福新锦州做出新的更大的贡献！
